# Germline Variants in Driver Genes of Breast Cancer and Their Association with Familial and Early-Onset Breast Cancer Risk in a Chilean Population

**DOI:** 10.3390/cancers12010249

**Published:** 2020-01-20

**Authors:** Alejandro Fernandez-Moya, Sebastian Morales, Trinidad Arancibia, Patricio Gonzalez-Hormazabal, Julio C. Tapia, Raul Godoy-Herrera, Jose Miguel Reyes, Fernando Gomez, Enrique Waugh, Lilian Jara

**Affiliations:** 1 Programa de Genética Humana, Instituto de Ciencia Biomédicas (ICBM), Facultad de Medicina, Universidad de Chile, Santiago 8380453, Chile; alefernandez@ug.uchile.cl (A.F.-M.); triniar17@gmail.com (T.A.); patriciogonzalez@uchile.cl (P.G.-H.); rgodoy@med.uchile.cl (R.G.-H.); 2 Laboratorio de Transformación Celular, Departamento de Oncología Básico Clínica, Facultad de Medicina, Universidad de Chile, Santiago 8380453, Chile; jtapia@med.uchile.cl; 3 Clínica Las Condes, Santiago 7591047, Chile; jmreyes@clc.cl; 4 Clínica Santa María, Santiago 7520378, Chile; fgomez@csm.cl (F.G.); ewaugh@csm.cl (E.W.)

**Keywords:** genetic predisposition to breast cancer, breast cancer risk, driver genes, germline variants, single nucleotide polymorphisms

## Abstract

The genetic variations responsible for tumorigenesis are called driver mutations. In breast cancer (BC), two studies have demonstrated that germline mutations in driver genes linked to sporadic tumors may also influence BC risk. The present study evaluates the association between SNPs and SNP-SNP interaction in driver genes *TTN* (rs10497520), *TBX3* (rs2242442), *KMT2D* (rs11168827), and *MAP3K1* (rs702688 and rs702689) with BC risk in *BRCA1/2*-negative Chilean families. The SNPs were genotyped in 489 BC cases and 1078 controls by TaqMan Assay. Our data do not support an association between rs702688: A>G or rs702689: G>A and BC risk. The rs10497520-T allele was associated with a decreased risk in patients with family history of BC or early-onset BC (OR = 0.6, *p* < 0.0001 and OR = 0.7, *p* = 0.05, respectively). rs2242442-G was associated with a protective effect and rs11168827-C was associated with increased BC risk in families with a strong history of BC (OR = 0.6, *p* = 0.02 and OR = 1.4, *p* = 0.05, respectively). As rs10497520-T and rs2242442-G seemed to protect against BC risk, we then evaluated their combined effect. Familial BC risk decreased in a dose-dependent manner with the protective allele count, reflecting an additive effect (*p*-trend < 10^−4^). To our knowledge, this is the first association study of BC driver gene germline variations in a Chilean population.

## 1. Introduction

In females, breast cancer (BC) has the highest incidence of any cancer worldwide. At least 1.15 million patients are diagnosed annually, comprising about 23% of all cancer cases in women [1,2]. Roughly 1 in 8 women alive today will contract BC in their lifetimes [3]. Chile is no exception to these global statistics, as BC has the highest mortality rate among cancers in Chilean women. BC caused 1511 deaths in 2015 in this country, with a mortality rate of 16.6 per 100,000 [4,5]. BC incidence is also on the rise nationally [5,6].

Identification of the tumor suppressor genes *BRCA1* (MIM 113705) [7] and *BRCA2* (MIM 600185) [8,9] spurred significant progress in understanding the genetic etiology of BC. Mutations in these two genes are considered to be high-penetrance BC susceptibility variations [2,10]. Studies suggest that about 16–20% of familial BC risk is attributable to *BRCA1/2* variants [11,12,13]. It is very likely that moderate-or low-penetrance susceptibility alleles are responsible for a large proportion of BC cases in families that do not carry *BRCA1/2* mutations [14]. As alluded, susceptibility mutations can be categorized as high-, moderate-, or low-penetrance according to the associated risk of developing BC [15]. All known BC susceptibility genes account for about half of hereditary BC (HBC) cases [11]; the genes responsible for the remaining half are yet to be determined. Identifying new BC susceptibility genes or alleles will improve risk assessment, shed light onto cancer mechanisms, and enhance the effectiveness of treatment.

The genomes of all cancers contain somatic mutations. Driver mutations are a subgroup of such variations that are causally involved in oncogenesis, as they confer cancer cells with a clonal selective advantage [16]. The remaining variations are called passenger mutations. A typical tumor contains 2–8 driver mutations. Although the specific driver mutations and mutational processes underlying BC have yet to be comprehensively probed [17], about 90% of BC tumors may be the result of somatic driver mutations that trigger the carcinogenic process [16,18,19]. Most known driver genes were identified in sporadic breast tumors using Next Generation Sequencing (NGS), including *ARID1B, CASP8*, *MAP3K1*, *MAP3K13*, *NCOR1*, *SMARCD1*, *CDKN1B*, *AKT2*, and *TBX3*. These genes contain low-frequency driver mutations, according to the gene databases ClinVar and dbSNP. Researchers have recently begun to explore whether the driver genes in sporadic tumors might also contain heritable variants associated with cancer risk. Göhler et al. (2017) [20] demonstrated an association between germline variants in the driver genes of sporadic cancer and BC risk, tumor characteristics and/or survival in a Swedish cohort with BC. These authors also studied a set of single-nucleotide polymorphisms (SNPs) in 15 genes commonly categorized as BC driver genes according to NGS analysis, identifying five genes with a potential link to BC susceptibility. (1) *TBX3* (rs2242442): The minor allele for this SNP correlated with decreased BC risk (OR = 0.76 [95% CI = 0.64–0.92], *p* = 0.004). (2) *TTN* (rs10497520): Homozygosity for the minor allele was associated with increased BC risk (OR = 1.96 [95% CI = 1.18–3.26], *p* = 0.001). (3) *MAP3K1* (rs702688 and rs72758040): Homozygosity for these SNPs correlated with increased risk (OR = 1.33 [95% CI = 0.99–1.76], *p* = 0.05 and OR = 1.36 [95% CI = 1.01–1.83], *p* = 0.04), respectively). (4) *KMT2D* (rs11168827): This SNP correlated with increased BC risk (OR = 1.31 [95% CI = 1.00–1.72], *p* = 0.05) and was associated with positive hormone receptor status and low-grade tumors. (5) *SF3B1* (rs4688): The minor allele correlated with decreased BC risk (OR = 0.75 [95% CI = 0.54–0.97], *p* = 0.03). This SNP was also associated with negative lymph node findings, metastases, and hormone receptor status [20]. To date, the mutations and variants in these novel driver genes have not been studied in a Chilean or Latin American population, and it remains unknown whether inherited variants in the driver genes affect cancer risk. Genetic variations typically vary by ethnicity, meaning that findings for one group may not applicable to Chilean or other populations.

The present study evaluates the association between specific SNPs and SNP-SNP interactions in the driver genes *TTN*, *TBX3*, *KMT2D*, and *MAP3K1* with familial and early-onset non-familial BC in Chilean families who are negative for *BRCA1/2* point mutations. A case-control study was used to explore the relationship between BC susceptibility and the following SNPs: s702688 and rs702689 (*MAP3K1*), rs2242442 (*TBX3*), rs10497520 (*TTN*), and rs11168827 (*KMT2D*). Moreover, we carried out a SNP-SNP interaction between rs2242442 and rs10497520 to evaluate their combined effect on the BC risk.

## 2. Results

### 2.1. Association Study between rs10497520, rs2242442, rs11168827, rs702688 and rs702689 with Familial Breast Cancer and Early-Onset Non-Familial Breast Cancer in Non-Carriers of BRCA1/2 Mutations

The cases were divided into two subgroups for the case-control analysis according to family history: Subgroup A (two or more family members with breast/ovarian cancer, *n* = 311) and Subgroup B (non-familial early-onset (diagnosis at ≤50 years of age) BC, *n* = 178). Table 1 shows the genotype and allele frequencies of the rs10497520:C>T (*TTN*), rs2242442:G>A (*TBX3*), rs11168827:G>A (*KMT2D*), and rs702688:A>G and rs702689:G>A (*MAP3K1*) polymorphisms in the whole data set, subgroups A and B, and controls. The genotype frequencies were in Hardy-Weinberg equilibrium for four of the five polymorphisms in controls (*p* = 0.69 for rs2242442:G>A, *p* = 0.30 for rs11168827:G>A, *p* = 0.74 for rs702688:A>G, and *p* = 0.75 for rs702689:G>A, respectively), while the *p*-value was 0.03 for rs10497520:C>T.

In the single-locus analyses, no significant differences were detected for rs702688:A>G or rs702689:G>A (both located in the *MAP3K1* gene) genotype or allele distributions, either in the whole dataset or subgroups A or B (*p* > 0.05).

For rs10497520:C>T (located in the *TTN* gene), the genotype and allele distribution was significantly different in the whole sample of BRCA1/2-negative cases and subgroup A as compared to controls (*p* ≤ 0.05) (Table 1). The minor allele frequency (MAF) (allele T) was significantly lower in the whole BC sample (39.7%), subgroup A (38.4%), subgroup B (41.9%) vs. control (47.5%) (OR = 0.7 [95% CI = 0.6–0.8], *p* < 0.0001, OR = 0.6 [95% CI = 0.5–0.8], *p* < 0.0001, and OR = 0.7 [95% CI = 0.6–0.9], *p* = 0.05, respectively) (Table 1). This result indicates that the T allele is associated with a protective effect against BC risk. We also observed a protective effect for T/T homozygosity in the whole sample (OR = 0.5 [95% CI = 0.3–0.9], *p* < 0.0001), subgroup A (OR = 0.4 [95% CI = 0.3–0.7], *p* = 0.0001), and subgroup B (OR = 0.6 [95% CI = 0.3–0.9], *p* = 0.05) (Table 1). Moreover, the distribution of T allele carriers (C/T + T/T) was significantly lower in the whole BC sample (OR = 0.6 [95% CI = 0.5–0.8], *p* = 0.001) and subgroup A (OR = 0.6 [95% CI = 0.4–0.8], *p* = 0.0009) vs. control, again indicating a protective effect of the T allele (Table 1). We then assessed for a protective effect of rs10497520:C>T according to number of BC cases per family (Table 2). BC risk was significantly decreased in homozygous T/T and T allele carriers (C/T + T/T) in families with two BC/OC cases (OR = 0.4 [95% CI = 0.2–0.7], *p* = 0.001 and OR = 0.6 [95% CI = 0.4–0.8], *p* = 0.01, respectively). Similarly, there was a protective effect for homozygous T/T and T allele carriers (C/T + T/T) in families with a strong family history of BC (OR = 0.5 [95% CI = 0.3–0.9], *p* = 0.01 and OR = 0.6 [95% CI = 0.4–0.9], *p* = 0.01, respectively). These results consistently suggest that the T allele was associated with a protective effect in Chilean *BRCA1/2*-negative families.

The genotype and allele distributions did not differ significantly between cases and controls for rs2242442:G>A (located in the *TBX3* gene) in either the whole-group or subgroup analysis (*p* > 0.05) (Table 1). However, when we analyzed the effect of rs2242442:G>A according to number of BC cases per family, we found that heterozygous A/G and G allele carriers (G/A + A/A) had a significantly decreased BC risk (OR = 0.6 [95% CI = 0.4–0.9], p=0.03 and OR = 0.6 [95% CI = 0.4–0.9], *p* = 0.02, respectively), indicating that the G allele is associated with a protective effect in the families with strong history of BC (Table 2).

In the case-control analysis, no significant differences were observed for genotype or allele distribution for rs11168827:G>C (located in the *KMT2D* gene), in the whole BC sample or subgroup A or B vs. controls (*p* > 0.05) (Table 1). However, BC risk was significantly elevated in heterozygous G/C individuals that had three or more family members with BC/OC (OR = 1.4 [95% CI = 1.0–2.1], *p* = 0.05) (Table 2). This result reflects and association between the C allele and BC risk in families with a strong history of BC.

### 2.2. Combined Effect between TTN rs10497520-T and TBX3 rs2242442-G Alleles with Breast Cancer Risk

As *TTN* and *TBX3* are driver or potential driver genes, and rs10497520-T and rs2242442-A seem to protect against BC risk, we evaluated the combined effects of these variants. For this analysis, cases were divided into five groups according to risk allele count: zero (G/G + C/C), one (G/G + C/T, G/A + C/C), two (G/G + T/T, A/A + C/C, G/A + C/T), three (G/A + T/T, A/A + C/T), or four (A/A + T/T). As shown in Table 3, the distributions of the combined genotypes in the whole BC sample and subgroup A differed significantly from the controls (global *p* 0.0003 and 0.0008, respectively), and BC risk decreased in a dose-dependent manner in the whole case group and subgroup A with the number of risk alleles (*p*-trend < 10^−4^ and <10^−4^, respectively). No additive effect was observed for early-onset BC (diagnosis ≤ 50 years of age). We also analyzed this additive effect according to number of BC cases per family (Table 4). A protective effect was found in the families with two BC/OC cases and families with the strongest history of BC (*p*-trend = 0.004 and 0.0007, respectively). These results indicate an additive effect of *TTN* rs10497520 and *TBX3* rs2242442 in the protection conferred.

## 3. Discussion

As there is widespread agreement that only about 16% of heritable breast and ovarian cancer risk is attributable to the high-penetrance *BRCA1/2* mutations [12,13], it seems likely that many BC cases in *BRCA1/2*-negative families could be attributable to moderate- or low-penetrance genes [14]. However, the sum total of BC susceptibility genes identified to date only explain about half of HBC incidence [11].

The driver mutations and mutational processes underlying BC have not yet been comprehensively explored [17]. Nevertheless, it has been proposed that around 90% of BC tumors are caused by somatic driver mutations that initiate the carcinogenic process [16,18,19]. Göhler et al. (2017) [20] investigated whether known driver genes may contain inherited variants in Swedish BC patients. To date, the article published by Göhler et al. [20] is the only study on germline variations in driver genes. In the discussion, the authors state that their results should be replicated in other populations. There have been no studies related to mutations or variants in driver genes in Chile or anywhere in Latin America, the following question, then, emerges: Could germline variations (SNPs) in driver genes influence BC risk in Chilean population? In the present study, we evaluated the impact of specific SNPs in the driver genes *TTN*, *TBX3*, *KMT2D*, and *MAP3K1* on familial and early-onset BC in Chilean families negative for *BRCA1/2* point mutations. To this end, we performed a case-control study to examine the association between BC risk and rs702688 and rs702689 (*MAP3K1*), rs2242442 (*TBX3*), rs10497520 (*TTN*), and rs11168827 (*KMT2D*).

The SNPs rs702689 and rs702688 are located in the coding region of *MAP3K1* gene [20]. The *MAP3K1* gene has been classified as a driver gene and acts within the MAP-signaling pathway, which triggers the expression of genes important for angiogenesis, proliferation, and cell migration [17]. Therefore, it is important to determine whether the SNPs rs702689 and rs702688 contribute to HBC risk in a Chilean population. Our data do not support an association between rs702688:A>G or rs702689:G>A and BC risk. With respect to rs702688:A>G, our results diverge from those reported by Göhler et al., who showed an elevated BC risk in individuals homozygous for the minor allele of rs702688 (A/A) [20]. To date, the Göhler et al. [20] study constitutes the only publication to evaluate the association between rs702688:A>G and HBC risk. G is the minor allele in Chilean and other Latin American populations. The control frequencies of rs702688-A (56.4%) and rs702688-G (43.6%) in this Chilean population are similar to those reported in the Ensembl database for Latin American control populations (57% for rs702688-A and 43% for rs702688-G). Therefore, it is possible that the rs702688 SNP is not associated with BC risk in Latin Americans. Regarding rs702689:G>A, there are no data in the literature on the association between this SNP and hereditary or sporadic BC risk.

The T-box transcription factor 3 gene (*TBX3*) belongs to a gene family that shares a common DNA-binding domain, the T-box. T-box genes encode transcription factors involved in regulating developmental processes. *TBX3* is expressed in mammary tissues and plays a context-dependent role in mammary gland development as well as in tumorigenesis [21]. TBX3 interacts with several major oncogenic pathways and is overexpressed in many tumors, including BC [22]. Recently, somatic variations in *TBX3* have been classified as BC driver mutations [17,23,24,25,26]. Marouf et al. [27] investigated the rs2242442 germline variation in a Moroccan population, finding that the homozygous genotype A/A was associated with elevated BC risk (OR = 3.93 [95% CI = 1.84–8.42], *p* = 0.0004). Nevertheless, Göhler et al. [20] showed that rs2242442 A allele carriers have a significantly decreased BC risk (OR = 0.76 [95% CI = 0.64–0.92], *p* = 0.004) in a Swedish population. The previously-cited articles are only studies that have conducted association analyses for rs2242442 and BC risk. Our results shown that the rs2242442 A allele has a protective effect in families with a strong family history of BC (≤3 BC cases), in agreement with the findings obtained by Göhler et al.

*TTN* (titin or connectin), the largest polypeptide encoded by the human genome, is a protein more generally known for its structural and elastic roles in muscle contractile machinery [28]. However, it has been suggested that *TTN* also has a critical role in establishing or maintaining chromosome compaction. Analogous to its role in muscle, TTN may localize to chromosomes and provide a template for the correct binding and assembly of other proteins involved in chromosome condensation [29]. Therefore, *TTN* mutations could affect the condensation and segregation of chromosomes, playing an important role in oncogenesis. Göhler et al. [20] described six SNPs in *TTN* that are associated with increased BC risk, aggressive tumor characteristics, and/or poor survival; of relevance to the present findings, homozygosity for the minor allele of rs10497520:C>T was associated with BC risk (OR = 1.96 [95% CI = 1.18–3.26], *p* = 0.01) in a Swedish population. In contrast, our results showed that the rs10497520-T allele, T/T homozygosity, or carrying the T allele (C/T + T/T) had a protective effect in BRCA1/2-negative Chilean women with a strong family history BC or non-familial early-onset BC, with highly significant p-values. One important issue to consider is that the genotype distribution of rs10497520 was in Hardy-Weinberg disequilibrium in our study, which could distort the results. The possibility that different selective factors may directly or indirectly alter the association between rs10497520 and BC risk cannot be discarded.

It has been reported that KMT2D is part of the histone methyltransferase (HMT) complex that directs tri-methylation of histone H3 lysine 4. These chromatin modifications stimulate transcriptional activation of target genes [30]. KMT2D has been shown to be involved in several cellular signaling pathways, regulating different sets of genes. A possible role for *KMT2D* as a tumor suppressor gene has also been proposed [31]. rs11168827, located in the *KMT2D* gene, was associated with BC risk (OR = 1.31 [95% CI = 1.00–1.72], *p* = 0.05), positive hormone receptor status, and low-grade tumors in a Swedish population. Our results are consistent with these findings, as we found that G/C heterozygosity was associated with elevated BC risk (OR = 1.4 [95% CI = 1.0–2.1], *p* = 0.05) in Chilean women with a strong family history of BC. Although our study provides evidence for an association of rs2242442 (*TBX3)*, rs10497520 (*TTN*) and rs11168827 (*KMT2D*) with BC risk, certain limitations must be considered. Firstly, the genotype distribution of rs10497520 did not conform to the Hardy–Weinberg expectations (*p* = 0.03), which may distort the results. Secondly, the sample size of the whole group in the present study is sufficient to yield 80% power; nevertheless, the sample size limits the subgroup analyses. Therefore, these results should be replicated using subgroups with larger sample sizes.

**Figure 1:**
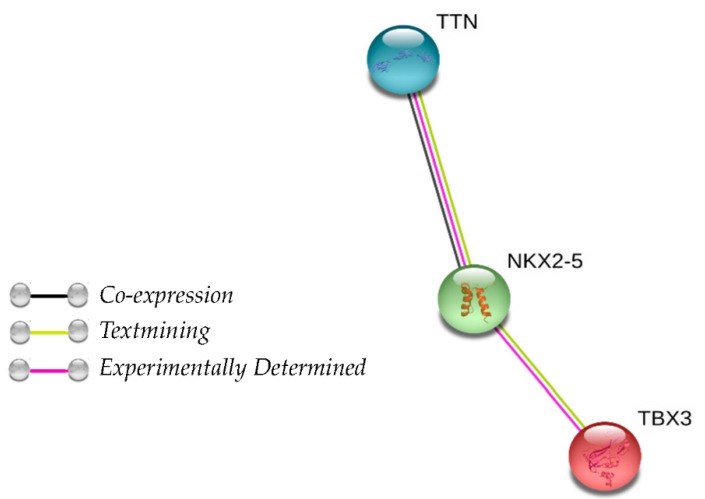
Protein association network in STRING analysis showing interactions of TBX3, NKX2-5, and TTN. Line colors indicate the mode of action of interaction between proteins.

As our results showed that the SNPs rs10497510-T (*TTN*) and rs2242442-A (*TBX3*) were associated with a protective effect, we evaluated their combined effect and constructed a genetic score based on the protective allele count. A dose-response association was observed for familial BC (Table 4). Several studies have demonstrated that *TTN* is highly mutated in several cancers, including BC, where the average mutation rate is 15.78% [32,33]. *TBX3* is a transcription factor frequently overexpressed in various types of human cancers, especially breast cancer [21]. There is no information in the literature regarding the interaction between the two genes. Nevertheless, it is possible that the SNP rs10497520-T increases chromosome compaction and rs2242442-A produces down-expression of specific genes; therefore, both SNPs could increase the protective effect. In order to assess whether there is an interaction between TBX3 and TTN proteins that could explain a synergistic protective effect, we used STRING software v11.0 (https://string-db.org/) to analyze the protein-protein interaction between TTN-TBX3. We found that TTN related indirectly to TBX3 through NKX2-5, which is an homeobox gene (Figure 1). Further studies are necessary to evaluate the functional impact of rs10497520-T (*TTN*) and rs2242442-G (*TBX3*) in the BC tumorigenesis.

Finally, it is important to note that the literature on the SNPs rs10497520:C>T (*TTN*), rs2242442:G>A (*TBX3*), rs11168827:G>A (*KMT2D*), and rs702688:A>G and rs702689:G>A (*MAP3K1*) is sparse; for the majority of these SNPs, the only study to date has been the Göhler et al. [20] report, making our data the first available for a Latin American population. Our results in Chilean population differ markedly from those obtained in the Swedish study, possibly due to the ethnic composition of the Chilean population. The contemporary Chilean population was produced by an admixture of Amerindian peoples with sixteenth- and seventeenth-century Spanish settlers. Later (nineteenth-century) immigration from Germany, Italy, Croatia, and Middle Eastern nations had a negligible effect on the ethnic makeup of the country (representing less than 4% of the national population), and any impact was largely circumscribed to the localities where the immigrants were concentrated [34]. The relationships among ethnicity, Amerindian admixture, genetic markers, and socioeconomic strata in Chile are well documented [35,36]. Given that the Chilean population is ~52% Caucasian and ~44% Native American, studies in other populations are needed to explore the general applicability of these findings [37].

## 4. Materials and Methods

### 4.1. Families

We selected 489 BC patients from 489 *BRCA1/2*-negative Chilean families at high risk for BC from records provided by the Servicio de Salud del Área Metropolitana de Santiago, Corporación Nacional del Cáncer (CONAC) and other private healthcare centers in Santiago (Metropolitan Region). Index cases were screened for *BRCA1* and *BRCA2* mutations as previously described [38], and the index case with the highest likelihood of carrying a deleterious mutation was used to develop the pedigree for each family. All families were negative for Li-Fraumeni, ataxia-telangiectasia, Cowden disease, and other syndromes associated with BC.

All study families were of exclusively Chilean ancestry for at least the past 3 generations according to self-report and in-depth interviews with several family members from different generations. The family history for the sample relevant to the inclusion criteria is shown in Table 5. Notably, 18% (88/489) had cases of bilateral BC; 58% (284/489) had cases of both BC and ovarian cancer (OC); and 1.1% (5/489) had BC cases in males. Among the cases, mean age at diagnosis was 42.1 years, and 75.2% were diagnosed before 50 years of age.

The study was approved by the Institutional Review Board of the University of Chile School of Medicine (Project Code 1150117.1 March 2015). Informed consent was obtained from all participants.

### 4.2. Control Population

The control group of healthy Chilean individuals (*n* = 1078) was selected from CONAC files. Controls were not related to the study families and had no personal or significant family history of cancer according to an interview carried out by a geneticist in our research group. Over 90% of controls lived in Santiago. Anonymous DNA samples were obtained from the controls. All participants provided informed consent, and samples were obtained in compliance with applicable ethical and legal norms. The control sample was matched to the cases for age and socioeconomic strata.

### 4.3. Mutation Analysis

Genomic DNA was extracted from the peripheral blood lymphocytes of 1078 controls and 489 cases from the high-risk families. The sampling procedure was performed as described by Chomczynski and Sacchi [39].

The SNPs rs10497520 (C>T), rs2242442 (G>A), rs11168827 (G>A), rs702688 (A>G) and rs702689 (G>A) were genotyped using commercially-available TaqMan Genotyping Assays (Thermo Fisher Scientific, Applied Biosystems, Waltham, MA, USA) (assay ID C__1958912_10, C__16174320_10, C__2023793_20, C__8961459_10 and C__8961434_10 respectively). The reaction was carried out in a 10 μL final volume containing 5 ng of genomic DNA, 1X TaqMan Genotyping Master Mix, and 20X TaqMan SNP Genotyping Assay. Polymerase chain reaction (PCR) was performed in a StepOnePlus Real-Time PCR System (Applied Biosystems, Foster City, CA, USA). The thermal cycles were as follows: 10 min at 95 °C then 40 cycles at 92 °C for 15 s and 60 °C for 1 min. Each genotyping run contained control DNA confirmed by sequencing. The alleles were assigned using StepOne software, v2.2 (Applied Biosystems). As a quality control, we repeated the genotyping on ~10% of the samples, and all genotype scoring was performed and checked separately by two reviewers blind to case-control status.

### 4.4. Statistical Analysis

The control data was assessed for Hardy-Weinberg equilibrium using a goodness-of-fit chi-square test (HW Chisq function, “Hardy Weinberg” package v1.4.1). Fisher’s exact test was used to test the association between genotypes/alleles and case/control status. Odds ratios (OR) with 95% confidence intervals (CI) were calculated to estimate the strength of the associations (odds ratios and Fisher’s exact test functions were performed using GraphPad Prism software v6.0 for Windows 10, Graphpad Software, La Jolla, CA, USA, www.graphpad.com). The cutoff for significance was a two-tailed *p*-value ≤ 0.05. The Cochran-Armitage trend test was performed to test the additive genetic effect model (CATT function in ‘Rassoc’ package v1.03 for R, Foundation for Statistical Computing, Vienna, Austria, https://www.r-project.org/). A chi-square test for trend was performed to test for additive effects of the SNPs (‘*p*-trend’ was determined in the Stata/MP v13.0 for Windows 10, Unix-StataCorp, College Station, TX, USA; ‘*p*-trend’ package).

## 5. Conclusions

Our study suggests that germline variants in driver genes *TTN* (rs10497520), *TBX3* (rs2242442) and *KMT2D* (rs11168827) can influence BC risk in *BRCA1/2*-negative Chilean families. Moreover, the presence of rs10497520 and rs2242442 could increase the protector effect of BC risk in Chilean population. To our knowledge, this is the first association study between germline variants in driver genes and BC risk in a South American population; therefore, studies in other populations are needed in order to understand how germline variants in driver genes can impact BC risk. On the other hand, functional studies are needed to determine the biological impact of this variants.

## Figures and Tables

**Table 1:** Genotype and allelic frequencies of rs10497520 (*TTN*), rs2242442 (*TBX3*), rs11168827 (*KMT2D*), rs702688 and rs702689 (*MAP3K1*) in *BRCA1/2*-negative breast cancer cases and controls.

Genotype or Allele	Controls (%) (*n* = 1078)	All BC Cases (*n* = 489)			Families with ≥2 BC and/or OC Cases (*n* = 311)			A Single case, Diagnosis at ≤50 Years of Age (*n* = 178)		
		BC Cases (%)	OR [95% CI]	*p*-Value ^a^	BC Cases (%)	OR [95% CI]	*p*-Value ^a^	BC Cases (%)	OR [95% CI]	*p*-Value ^a^
rs10497520 (*TTN*)										
C/C	314 (29.1)	182 (37.2)	1.0 (Ref)	-	122 (39.2)	1.0 (ref)	-	60 (33.7)	1.0 Ref	-
C/T	504 (46.8)	226 (46.2)	**0.7 [0.6–0.9]**	**0.04**	139 (44.7)	0.7 [0.5–0.9]	0.02	87 (48.9)	0.9 [0.6–1.2]	0.58
T/T	260 (24.1)	81 (16.6)	**0.5 [0.3–0.9]**	**<0.0001**	50 (16.1)	**0.4 [0.3–07]**	**0.0001**	31 (17.4)	**0.6 [0.3–0.9]**	**0.05**
C/T + T/T	764 (70.9)	307(62.8)	**0.6 [0.5–0.8]**	**0.001**	189 (60.8)	**0.6 [0.4–0.8]**	**0.0009**	118 (66.3)	0.8 [0.5–1.1]	0.21
Allele C	1132 (52.5)	590 (60.3)	1.0 (Ref)	-	383 (61.6)	1.0 (ref)	-	207 (58.1)	1.0 (Ref)	-
Allele T	1024 (47.5)	388 (39.7)	**0.7 [0.6–0.8]**	**<0.0001**	239 (38.4)	**0.6 [0.5–0.8]**	**<0.0001**	149 (41.9)	**0.7 [0.6–0.9]**	**0.05**
rs2242442 (TBX3)										
G/G	674 (62.5)	328 (67.1)	1.0 (Ref)	-	210 (67.5)	1.0 (Ref)	-	118 (66.3)	1.0 (Ref)	-
G/A	358 (33.2)	146 (29.9)	0.8 [0.6–1.0]	0.14	90 (28.9)	0.8 [0.6–1.0]	0.14	56 (31.5)	0.8 [0.6–1.2]	0.54
A/A	46 (4.3)	15 (3.0)	0.6 [0.3–1.1]	0.20	11 (3.5)	0.7 [0.3–1.5]	0.52	4 (2.2)	0.4 [0.1–1.4]	0.21
G/A + A/A	404 (37.5)	161 (32.9)	0.8 [0.6–1.0]	0.08	101 (32.5)	0.8 [0.6–1.0]	0.10	60 (33.7)	0.8 [0.6–1.1]	0.35
Allele G	1706 (79.1)	802 (82.0)	1.0 (Ref)	-	510 (82.0)	1.0 (Ref)	-	292 (82.0)	1.0 (Ref)	-
Allele A	450 (20.9)	176 (18.0)	0.8 [0.6–1.0]	0.06	112 (18.0)	0.8 [0.6–1.0]	0.13	64 (18.0)	0.8 [0.6–1.1]	0.23
rs11168827 (*KMT2D*)										
G/G	439 (40.7)	198 (40.5)	1.0 (ref)	-	121 (38.9)	1.0 (ref)	-	77 (43.3)	1.0 (ref)	-
G/C	510 (47.3)	239 (48.9)	1.0 [0.8–1.3]	0.77	157 (50.5)	1.1 [0.8–1.4]	0.45	82 (46.1)	0.9 [0.6–1.3]	0.66
C/C	129 (12.0)	52 (10.6)	0.8 [0.6–1.2]	0.58	33 (10.6)	0.9 [0.6–1.4]	0.82	19 (10.7)	0.8 [0.4–1.4]	0.59
G/C + C/C	639 (59.3)	291 (59.5)	1.0 [0.8–1.2]	0.95	190 (61.1)	1.0 [0.8–1.3]	0.59	101 (56.7)	0.9 [0.6–1.2]	0.56
Allele G	1388 (64.4)	635 (64.9)	1.0 (ref)	-	399 (64.1)	1.0 (ref)	-	236 (66.3)	1.0 (ref)	-
Allele C	768 (35.6)	343 (35.1)	0.9 [0.8–1.1]	0.79	223 (35.9)	1.0 [0.8–1.2]	0.95	120 (33.7)	0.9 [0.7–1.1]	0.52
rs702688 (*MAP3K1*)										
A/A	345 (32.0)	167 (34.2)	1.0 (Ref)	-	100 (32.3)	1.0 (Ref)	-	67 (37.6)	1.0 (Ref)	-
A/G	525 (48.7)	236 (48.3)	0.9 [0.7–1.1]	0.58	150 (48.6)	0.9 [0.7–1.3]	0.94	85 (47.8)	0.8 [0,.5–1.1]	0.32
G/G	208 (19.3)	86 (17.6)	0.8 [0.6–1.1]	0.34	60 (19.3)	0.9 [0.6–1.4]	1.0	26 (14.6)	0.6 [0.3–1.0]	0.08
A/G + G/G	733 (68.0)	322 (65.8)	0.9 [0.7–1.1]	0.41	210 (67.8)	0.9 [0.7–1.3]	0.94	111 (62.4)	0.7 [0.5–1.0]	0.14
Allele A	1215 (56.4)	570 (58.3)	1,0 (Ref)	-	350 (56.4)	1.0 (Ref)	-	219 (61.5)	1.0 (Ref)	-
Allele G	941 (43.6)	408 (41.7)	0.9 [0.7–1.0]	0.33	270 (43.6)	0.9 [0.8–1.1]	0.99	137 (38.5)	0.8 [0.6–1.0]	0.07
rs702689 (*MAP3K1*)										
G/G	274 (25.4)	132 (27.0)	1.0 (Ref)	-	82 (26.4)	1.0 (Ref)	-	48 (27.0)	1.0 (Ref)	-
G/A	544 (50.5)	226 (46.2)	0.8 [0.6–1.1]	0.28	133 (42.8)	0.8 [0.5–1.1]	0.22	92 (51.7)	0.9 [0.6–1.4]	0.84
A/A	260 (24.1)	131 (26.8)	1.0 [0.7–1.4]	0.82	96 (30.9)	1.2 [0.8–1.7]	0.26	38 (21.3)	0.8 [0.5–1.3]	0.48
G/A + A/A	804 (74.6)	357 (73.0)	0.9 [0.7–1.1]	0.53	229 (73.6)	0.9 [0.7–1.2]	0.76	130 (73.0)	0.9 [0.6–1.3]	0.64
Allele G	1092 (50.6)	490 (50.1)	1.0 (Ref)	-	297 (47.7)	1.0 (Ref)	-	188 (52.8)	1.0 (Ref)	-
Allele A	1064 (49.4)	488 (49.9)	1.02 [0.8–1.1]	0.81	325 (52.3)	1.1 [0.9–1.3]	0.21	168 (47.2)	0.9 [0.7–1.1]	0.48

BC—breast cancer, OC—ovarian cancer, OR—odds ratio, CI—confidence interval, Ref—Reference; a Fisher’s exact test; Bold values are statistically significant (*p* < 0.05).

**Table 2:** Genotype and allelic frequencies of rs10497520 (*TTN*), rs2242442 (*TBX3*), rs11168827 (*KMT2D*), rs702688 and rs702689 (*MAP3K1*) according the number of BC cases in the families in *BRCA1/2*-negative breast cancer cases and controls.

Genotype or Allele	Controls (%) (*n* = 1078)	Families with 2 BC and/or OC Cases (*n* = 166)			Families with ≥3 BC and/or OC Cases (*n* = 145)		
		BC Cases (%)	OR [95% CI]	*p* Value ^a^	BC Cases (%)	OR [95% CI]	*p* Value ^a^
rs10497520 (*TTN*)							
C/C	314 (29.1)	65 (39.2)	1.0 (ref)	-	57 (39.3)	1.0 (ref)	-
C/T	504 (46.8)	77 (46.4)	0.7 [0.5–1.0]	0.11	62 (42.8)	**0.6 [0.4–0.9]**	**0.05**
T/T	260 (24.1)	24 (14.5)	**0.4 [0.2–0.7]**	**0.001**	26 (17.9)	**0.5 [0.3–0.9]**	**0.01**
C/T + T/T	764 (70.9)	101 (60.8)	**0.6 [0.4–0.8]**	**0.01**	88 (60.7)	**0.6 [0.4–0.9]**	**0.01**
Allele C	1132 (52.5)	207 (62.3)	1.0 (ref)	-	176 (60.7)	1.0 (ref)	-
Allele T	1024 (47.5)	125 (37.7)	**0.6 [0.5–0.8]**	**0.001**	114 (39.3)	**0.7 [0.5–0.9]**	**0.01**
rs2242442 (*TBX3*)							
G/G	674 (62.5)	105 (63.3)	1.0 (Ref)	-	105 (72.4)	1.0 (Ref)	-
G/A	358 (33.2)	54 (32.5)	0.9 [0.6–1.3]	0.92	36 (24.8)	**0.6 [0.4–0.9]**	**0.03**
A/A	46 (4.3)	7 (4.2)	0.9 [0.4–2.2]	1.00	4 (2.8)	0.5 [0.8–1.5]	0.38
G/A + A/A	404 (37.5)	61 (36.7)	0.9 [0.6–1.3]	0.93	40 (27.6)	**0.6 [0.4–0.9]**	**0.02**
Allele G	1706 (79.1)	264 (79.5)	1.0 (Ref)	-	246 (84.8)	1.0 (Ref)	-
Allele A	450 (20.9)	68 (20.5)	0.9 [0.7–1.2]	0.92	44 (15.2)	**0.6 [0.4–0.9]**	**0.02**
rs11168827 (*KMT2D*)							
G/G	439 (40.7)	72 (43.4)	1.0 (ref)	-	49 (33.8)	1.0 (ref)	-
G/C	510 (47.3)	74 (44.6)	0.8 [0.6–1.3]	0.53	**83 (57.2)**	**1.4 [1.0–2.1]**	**0.05**
C/C	129 (12.0)	20 (12.0)	0.9 [0.5–1.6]	0.89	13 (9.0)	0.9 [0.4–1.7]	0.87
G/C + C/C	639 (59.3)	94 (56.6)	0.9 [0.6–1.2]	0.55	96 (66.2)	1.3 [0.9–1.9]	0.12
Allele G	1388 (64.4)	218 (65.7)	1.0 (ref)	-	181 (62.4)	1.0 (ref)	-
Allele C	768 (35.6)	114 (34.3)	0.9 [0.7–1.2]	0.69	109 (37.6)	1.0 [0.8–1.4]	0.55
rs702688 (*MAP3K1*)							
A/A	345 (32.0)	51 (30.7)	1.0 (Ref)	-	49 (33.8)	1.0 (Ref)	-
A/G	525 (48.7)	83 (50.0)	1.0 [0.7–1.5]	0.77	68 (46.9)	0.9 [0.6–1.3]	0.68
G/G	208 (19.3)	32 (19.3)	1.0 [0.6–1.6]	0.90	28 (19.3)	0.9 [0.5–1.5]	0.90
A/G + G/G	733 (68.0)	115 (69.3)	1.0 [0.7–1.5]	0.78	96 (66.2)	0.9 [0.6–1.3]	0.70
Allele A	1215 (56.4)	185 (55.7)	1.0 (Ref)	-	166 (57.2)	1.0 (Ref)	-
Allele G	941 (43.6)	147 (44.3)	1.0 [0.8–1.2]	0.87	124 (42.8)	0.9 [0.7–1.2]	0.82
rs702689 (*MAP3K1*)							
G/G	274 (25.4)	41 (24.7)	1.0 (Ref)	-	41 (28.3)	1.0 (Ref)	-
G/A	544 (50.5)	71 (42.8)	0.8 [0.5–1.3]	0.52	62 (42.8)	0.7 [0.5–1.1]	0.22
A/A	260 (24.1)	54 (32.5)	1.3 [0.8–2.1]	0.14	42 (29.0)	1.0 [0.6–1.7]	0.81
G/A + A/A	804 (74.6)	125 (75.3)	1.0 [0.7–1.5]	0.92	104 (71.7)	0.8 [0.5–1.2]	0.47
Allele G	1092 (50.6)	153 (46.1)	1.0 (Ref)	-	144 (49.7)	1.0 (Ref)	-
Allele A	1064 (49.4)	179 (53.9)	1.2 [0.9–1.5]	0.13	146 (50.3)	1.0 [0.8–1.3]	0.79

BC—breast cancer, OC—ovarian cancer, OR—odds ratio, CI—confidence interval, Ref—Reference; a Fisher’s exact test; Bold values are statistically significant (*p* < 0.05).

**Table 3:** Combined effects of rs2242442 (*TBX3*) and rs10497520 (*TTN*) on the risk of breast cancer.

Number of Risk Alleles ^(a)^	Controls (*n* = 1078) (%)	All BC Cases (*n* = 489)			Families with ≥2 BC and/or OC Cases (*n* = 3 11)			A Single Case, Diagnosis at ≤50 Years of Age (*n* = 178)		
		BC Cases (%)	OR [95% CI]	*p* Value ^(b)^	BC Cases (%)	OR [95% CI]	*p* Value ^(b)^	BC Cases (%)	OR [95% CI]	*p* Value ^(b)^
0 risk alleles	200 (18.6)	125 (25.6)	1.0 (Ref)	-	84 (27.0)	1.0 (Ref)	-	41 (23.0)	1.0 (Ref)	-
1 risk allele	409 (37.9)	196 (40.1)	0.7 [0.5–1.0]	0.07	127 (40.8)	0.7 [0.5–1.0]	0.07	69 (38.8)	0.8 [0.5–1.2]	0.3
2 risk alleles	354 (32.8)	139 (28.4)	**0.6 [0.5–0.8]**	**0.002**	79 (25.4)	**0.5 [0.4–0.7]**	**0.0005**	60 (33.7)	0.8 [0.5–1.2]	0.4
3 risk alleles	103 (9.6)	29 (5.9)	**0.4 [0.2–0.7]**	**0.0007**	21 (6.8)	**0.4 [0.2–0.8]**	**0.006**	8 (4.5)	1.3 [0.5–3.1]	0.4
4 risk alleles	12 (1.1)	0 (0)	**0.06 [0.003–1.0]**	**0.004**	0 (0)	**0.09 [0.005–1.6]**	**0.02**	0 (0)	0.1 [0.01–3.3]	0.2
*P*-trend ^(c)^				**<10** ^**−4**^			**<10** ^**−4**^			**0.02**
Global *P* ^(d)^				**0.0003**			**0.0008**			0.08

^(a)^ 0 risk allele: G/G + C/C; 1 risk allele: G/G + C/T, G/A + C/C; 2 risk alleles: G/G + T/T, A/A + C/C, G/A + C/T; 3 risk alleles: G/A + T/T, A/A + C/T; 4 risk alleles: A/A + T/T; ^(b)^ Fisher’s exact test; ^(c)^ Chi-test for trend; ^(d)^ Chi-squared test for independence; BC—breast cancer; OC—ovarian cancer; OR—odds ratios, CI—confidence interval; Ref—Reference. *p* ≤ 0.05 Statistically significant (bold).

**Table 4:** Combined effects of rs2242442 (*TBX3*) and rs10497520 (*TTN*) on the risk of breast cancer according the number of BC cases in the families.

Number of Risk Alleles ^(a)^	Controls (*n* = 1078) (%)	Families with Two BC and/or OC Cases (*n* = 166)			Families with ≥3 BC and/or OC Cases (*n* = 145)		
		BC Cases (%)	OR [95% CI]	*p*-Value ^(b)^	BC Cases (%)	OR [95% CI]	*p*-Value ^(b)^
0 risk alleles	200 (18.6)	43 (25.9)	1.0 (Ref)	-	41 (28.3)	1.0 (Ref)	-
1 risk allele	409 (37.9)	67 (40.4)	0.7 [0.4–1.1]	0.1	60 (41.4)	0.7 [0.5–1.1]	0.2
2 risk alleles	354 (32.8)	**45 (27.1)**	**0.4 [0.2–0.7]**	**0.002**	**34 (23.4)**	**0.5 [0.3–0.9]**	**0.02**
3 risk alleles	103 (9.6)	**11 (6.6)**	**0.4 [0.2–0.9]**	**0.05**	**10 (6.9)**	**0.4 [0.2–1.0]**	**0.05**
4 risk alleles	12 (1.1)	0 (0.0)	0.1 [0.01–3.3]	0.2	0 (0.0)	0.1 [0.01–3.1]	0.2
*p*-trend^(c)^				**0.0007**			**0.004**
Global *p* ^(d)^				**0.01**			0.06

^(a)^ 0 risk allele: G/G + C/C; 1 risk allele: G/G + C/T, G/A + C/C; 2 risk alleles: G/G + T/T, A/A + C/C, G/A + C/T; 3 risk alleles: G/A + T/T, A/A + C/T; 4 risk alleles: A/A + T/T; ^(b)^ Fisher’s exact test; ^(c)^ Chi-test for trend; ^(d)^ Chi-squared test for independence; BC—breast cancer; OC—ovarian cancer; OR—odds ratios, CI—confidence interval; Ref—Reference. *p* ≤ 0.05 Statistically significant (bold).

**Table 5:** Inclusion criteria for the families included in this study.

Inclusion Criteria	Families *n* (%)
Three or more family members with breast and/or ovarian cancer	145 (29.7%)
Two family members with breast and/or ovarian cancer	166 (33.9%)
Single affected individual with breast cancer, onset ≤35 years of age	91 (18.6%)
Single affected individual with breast cancer, onset 36–50 years of age	87 (17.8%)
**TOTAL**	**489 (100%)**
